# Regulation of Oxalate Metabolism in Spinach Revealed by RNA-Seq-Based Transcriptomic Analysis

**DOI:** 10.3390/ijms22105294

**Published:** 2021-05-18

**Authors:** Vijay Joshi, Arianne Penalosa, Madhumita Joshi, Sierra Rodriguez

**Affiliations:** 1 Department of Horticultural Sciences, Texas A&M University, College Station, TX 77843, USA; 2 Texas A&M AgriLife Research and Extension Center, Uvalde, TX 78801, USA; Madhumita.Joshi@ag.tamu.edu; 3 College of Science, University of Texas, Arlington, TX 76019, USA; ariannecaileepenalosa@yahoo.com (A.P.); Sierra78881@yahoo.com (S.R.)

**Keywords:** oxalate, spinach, transcriptomics, isocitrate lyase

## Abstract

Although spinach (*Spinacia oleracea* L.) is considered to be one of the most nutrient-rich leafy vegetables, it is also a potent accumulator of anti-nutritional oxalate. Reducing oxalate content would increase the nutritional value of spinach by enhancing the dietary bioavailability of calcium and other minerals. This study aimed to investigate the proposed hypothesis that a complex network of genes associated with intrinsic metabolic and physiological processes regulates oxalate homeostasis in spinach. Transcriptomic (RNA-Seq) analysis of the leaf and root tissues of two spinach genotypes with contrasting oxalate phenotypes was performed under normal physiological conditions. A total of 2308 leaf- and 1686 root-specific differentially expressed genes (DEGs) were identified in the high-oxalate spinach genotype. Gene Ontology (GO) analysis of DEGs identified molecular functions associated with various enzymatic activities, while KEGG pathway analysis revealed enrichment of the metabolic and secondary metabolite pathways. The expression profiles of genes associated with distinct physiological processes suggested that the glyoxylate cycle, ascorbate degradation, and photorespiratory pathway may collectively regulate oxalate in spinach. The data support the idea that isocitrate lyase (*ICL*), ascorbate catabolism-related genes, and acyl-activating enzyme 3 (*AAE3*) all play roles in oxalate homeostasis in spinach. The findings from this study provide the foundation for novel insights into oxalate metabolism in spinach.

## 1. Introduction

The presence of oxalate is ubiquitous across the plant kingdom. Many plants belonging to the families *Chenopodiaceae*, *Amaranthaceae*, and *Polygonaceae* accumulate excessively high oxalate [1]. The functional significance of oxalate accumulation in plants can be attributed to several mechanisms, such as sequestering and regulating excess calcium [2,3,4,5,6,7], detoxifying heavy metals [8,9,10,11], protecting against insects [12,13] and diseases [14,15], and maintaining ionic balance [7]. Despite these multiple roles in plants, high oxalate levels in edible plant parts are a concern for human nutrition and health. Excess levels of oxalate in a regular diet significantly alter nutrient availability, as oxalate reacts with calcium and other minerals to form insoluble crystals, resulting in hyperoxaluria, a predominant risk factor for recurrent kidney stones [5,16,17]. Hence, in order to better understand its biological relevance in oxalogenic plants and manipulate its accumulation in edible plant parts, elucidating oxalate’s metabolic or gene regulatory mechanisms is essential.

Although spinach (*Spinacia oleracea* L.) is considered to be one of the most nutrient-rich leafy vegetables, it is also a potent accumulator of excessive amounts of anti-nutritional oxalate [18,19]. High consumption of spinach reduces calcium and magnesium absorption due to the formation of oxalate-bound insoluble complexes. Reducing the oxalic acid concentration would increase the nutritional value (by enhancing the dietary bioavailability of calcium and other minerals, ascorbates, and vitamins) and consumer acceptance (suppressing the bitter taste) of spinach. Strategies such as seasonal variations, genotypic differences, and modified agronomic techniques—such as nitrogen fertilizer management—have been proposed for the management the oxalate content of vegetables. Several studies have demonstrated increased oxalate accumulation with increasing amounts of available NO_3_^−^ in spinach [20,21,22] and rice [23]. Furthermore, manipulation of nitrogen forms or the ratio of NO_3_ to NH_4_ [24,25,26,27], along with seasonal and genetic effects [18], can also alter oxalate accumulation in spinach. Conflicting reports suggest both increases [28] and decreases [29] in oxalate concentrations with plant age in spinach. In general, oxalate content in spinach stems and petioles was lower than in leaves [26,30,31]. A study showing minimal correlation of oxalates with the leaf type (flat or savoy) and leaf weight of plants suggested the feasibility of the genetic improvement of spinach for oxalate reduction using high-yielding spinach varieties [19]. The presence of significant genetic variation in the amount of oxalic acid was first reported in a small population of 39 spinach breeding lines, hybrids, and F2 populations [32]. Demonstration of a converse relationship in fast-growing spinach cultivars that accumulated low oxalates but high nitrates, and vice versa, in a study comprising 182 open-pollinated and F1 hybrid cultivars and breeding lines [33], suggests the possibility of developing fast-growing cultivars as a breeding strategy to reduce oxalate accumulation. Although no specific genes were identified, an ethyl methanesulfonate (EMS)-mediated mutant in spinach [34] and ion-beam-mutagenized rice [35] accumulating lower oxalate levels suggests the possibility of the genetic improvement of spinach for oxalate reduction. Identification of SNP markers associated with high oxalate [36] in spinach has allowed accelerating efforts to breed spinach varieties with low oxalic acid content. Despite the wide range in oxalate concentrations observed among the genotypes, no major QTLs with significant impacts on oxalate concentration were identified, confirming the complexity of oxalate as a trait possibly regulated by several minor genes.

Oxalate biosynthesis in plants is complex, possibly involving five critical substrates—isocitrate, glycolate, glyoxylate, oxaloacetate, and ascorbate. Despite widespread occurrence across the plant kingdom, it remains unknown which specific pathways or genes directly or indirectly contribute to oxalate accumulation. Although oxalate production through photorespiratory glycolate/glyoxylate oxidation has been demonstrated [4,5,37,38,39], contradictory findings suggest the occurrence of glycolate-independent oxalate synthesis in plants [4,5,40]. The growing quantity of evidence using canonical and non-canonical pathways also implicates the role of ascorbates in oxalate synthesis [5,41,42,43,44,45,46,47]. Despite being naturally rich in oxalate, few efforts have been made to explore the precise contributions of these individual pathways to oxalate metabolism, expression of associated genes, or tissue specificities in spinach under normal physiological conditions. The only study performed on spinach, using a subset of putative genes involved in oxalate metabolism [31], suggested the roles of glycolate oxidases (*GLO/GXO*) and oxaloacetate acetyl hydrolases (*OXAC*) in oxalate synthesis. However, this study does not provide a detailed overview of global gene expression in the context of associated processes—such as photorespiration, or the glyoxylate cycle—or competing pathways sharing various substrates involved in the biosynthesis or catabolism of oxalate in spinach. We examined the global transcriptomic differences under normal physiological conditions in two spinach cultivars with contrasting oxalate phenotypes. For this study, we selected a pre-characterized high oxalate spinach accession, PI 175311 from the USDA germplasm repository [19] and a low oxalate heirloom spinach variety, ‘Bloomsdale’. The study was designed to test the hypothesis that a complex network of genes associated with intrinsic metabolic and physiological processes regulates the homeostasis of the endogenous oxalate in the spinach genotypes. The study identified differentially expressed transcripts in the leaf and root tissues of high-oxalate PI 175311. We have discussed the differential expression of the genes in the context of the known metabolic pathways associated with oxalate metabolism in plants. Our results provide a global view of the regulatory molecular mechanisms associated with oxalate accumulation in spinach. Additionally, the findings of this study could aid in mining genes regulating oxalate metabolism and engineering crops with reduced levels of oxalates.

## 2. Results

### 2.1. Validation of Contrasting Oxalate Contents in Spinach Genotypes

Little is known about how inherently high levels of oxalate could impact other physiological processes, or the plant’s nutrient status. Hence, a phenotypic characterization preceded the sampling of tissues for RNA-Seq analysis. The oxalate concentrations expressed in fresh and dry weight bases of the leaf laminae and roots of both of the genotypes showed significant differences (Table 1). PI 175311 accumulated 44% and 48% higher oxalate content in its leaves than Bloomsdale on a fresh and dry weight basis, respectively, validating the contrasting oxalate phenotypes. The oxalate content in roots was also 29% higher in PI 175311 than in Bloomsdale. The exchangeable cations (calcium, Ca; magnesium, Mg; and potassium, K) and total Kjeldahl nitrogen (TKN) content were significantly higher in the leaf laminae of PI 175311 (Table S1; Supplementary Materials). The gas exchange parameters in the leaves of 6-week-old spinach plants of both genotypes showed no apparent differences in the net photosynthetic rate, stomatal conductance, or transpiration rate, implying uncompromised photosynthetic performance (Table S2; Supplementary Materials).

### 2.2. Transcriptome Sequencing, Assembly and Quality Assessment

A total of 12 independent cDNA libraries—three each from the leaf laminae (XS1L1, XS1L2, and XS1L3 for PI 175311; XBDL1, XDL2, and XDL3 for Bloomsdale) and roots (XS1R1, XS1R2, and XS1R3 for PI 175311; XBDR1, XBDR2, and XBDR3 for Bloomsdale)—were subjected to the Illumina HiSeq platform. The raw reads were deposited in the Sequence Read Archive at the GeneBank database SRA ID: SRP252185 (Gene Expression Omnibus GSE146711). On average, 62.7 and 59.3 million raw reads were generated for leaf and root tissues, respectively, for PI 175311, while 56.8 and 59.4 million reads were generated for leaf and roots tissues, respectively, for Bloomsdale (Table S3; Supplementary Materials). On average, the Q20 and Q30 percentages across all reads were more than 98% and 95%, respectively (the sequencing error rate was less than 0.02%). Over 87% of reads were mapped to the reference genome (Table S4, Supplementary Materials) available at SpinachBase (http://www.spinachbase.org; accessed on 13 April 2019). On average, 86% of reads were uniquely mapped, while 4% were multi-mapped (Table S4) to the reference genome for both tissue types and genotypes. The high Pearson’s correlation among biological replicates (Figure S1; Supplementary Materials) confirmed the RNA-Seq data’s reliability. The gene expression levels among leaf and root tissues between the genotypes were comparable (Figure S2; Supplementary Materials). The results overall suggest high quality and coverage, providing a foundation for further analyses.

### 2.3. Analysis of Differentially Expressed Genes (DEGs) in Contrasting Oxalate Genotypes

**Figure 1:**
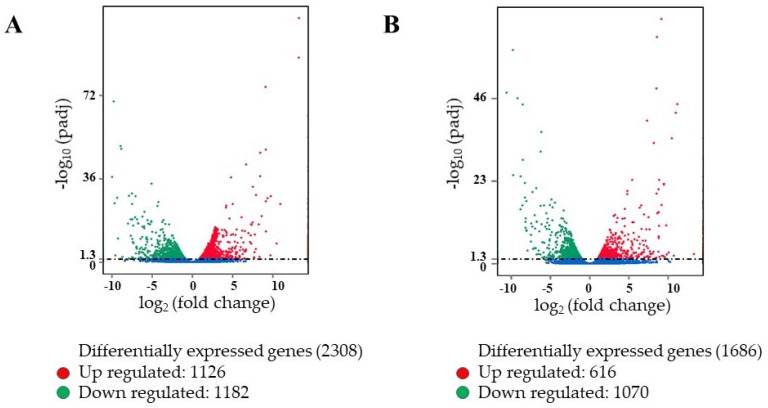
The volcano maps showing the number of differentially expressed genes in PI 175311 vs. Bloomsdale leaf (**A**) and root (**B**) tissues. Red dots represent upregulated genes, and green dots represent downregu-lated genes (*p* < 0.05). The *x*-axis shows the fold change of genes between different samples (padj < 0.05), and the *y*-axis coordinate indicates the statistically significant degree of changes in gene expression levels at −log10(padj *p*-value).

**Figure 2:**
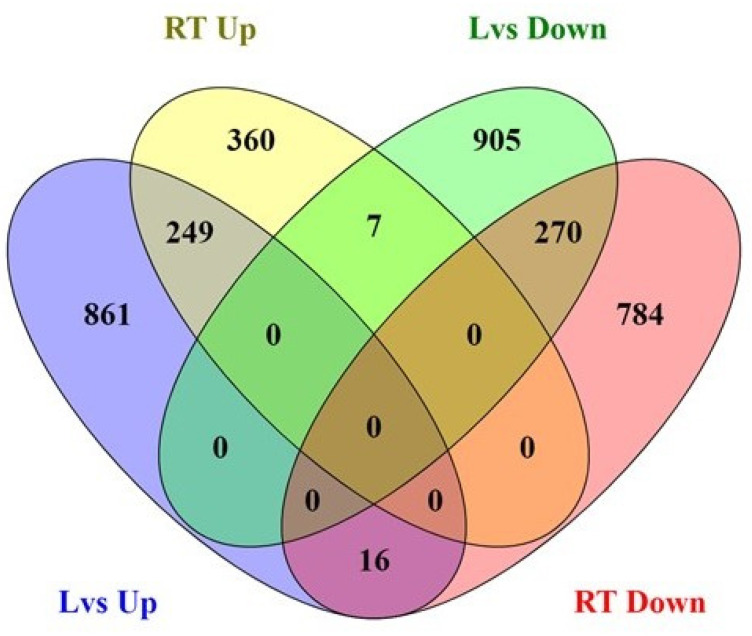
The Venn diagram shows the number of differentially expressed genes (DEGs) in each group and the overlaps between groups. (Lvs Up—Upregulated in leaf; RT Up—Upregulated in root; Lvs Down—Downregulated in leaf and RT Down—Downregulated in root). The number in the circle represents the total number of DEGs, and the overlap represents the DEGs in common.

The DEG analysis of leaf and root tissues identified 2308 and 1686 differentially expressed genes in the leaf and root tissues, respectively, of PI 175311 (Figure 1). The numbers of upregulated (1126) and downregulated (1182) genes were comparable in the leaf tissue (Tables S5 and S6; Supplementary Materials). On the other hand, in the root tissue, 616 transcripts were upregulated, while 1070 were downregulated (Tables S7 and S8; Supplementary Materials). As presented in the Venn diagram (Figure 2), 861 and 360 DEGs expressed in leaf and root tissues, respectively, were uniquely upregulated; meanwhile, 905 and 784 unique DEGs were downregulated in leaf and root tissues, respectively. Using the PlantTFDB database (http://planttfdb.gao-lab.org/; accessed on 15 April 2020), we identified 63 and 33 potential transcription factors (TFs) upregulated in leaf and root tissues, respectively (Figure S3; Supplementary Materials), while 77 and 72 TFs were downregulated in leaf and root tissues, respectively. Ten unique TFs were up- and downregulated in both leaf and root tissues. TFs from the MADS family [48] were prominent in leaf tissues, while bLBH members led in root tissues (Figure S4; Supplementary Materials).

### 2.4. Functional Annotation and Classification of Transcriptome

**Figure 3:**
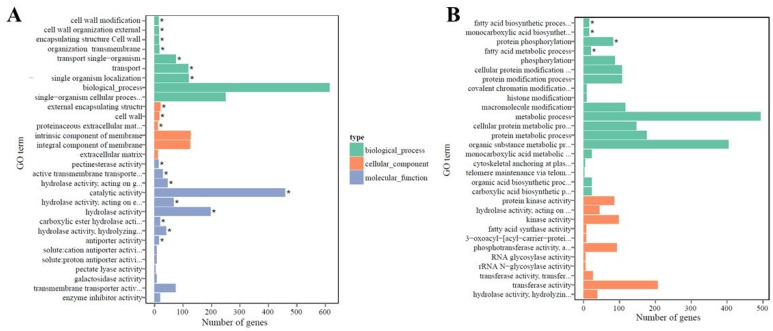
GO enrichment bar chart of DEGs: Gene Ontology (GO) of (**A**) upregulated and (**B**) downregulated differentially expressed genes (DEGs) in PI 175311 leaf tissue. The *y*-axis represents the enriched GO term, *x*-axis represents the number of DEGs enriched in the listed term. Colors represent different GO types: biological process, cellular component and molecular function. * GO with corrected *p*-value < 0.05 significantly enriched in DEGs.

**Figure 4:**
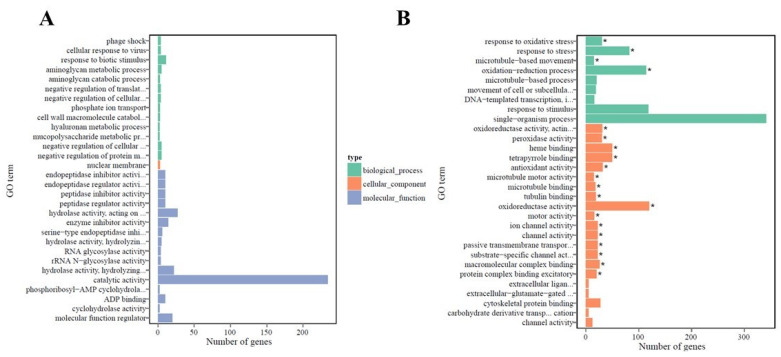
GO enrichment bar chart of DEGs: Gene Ontology (GO) of (**A**) upregulated and (**B**) downregulated differentially expressed genes (DEGs) in PI 175311 root tissue. The *y*-axis represents the enriched GO term, *x*-axis represents the number of DEGs enriched in the listed term. Colors represent different GO types: biological process, cellular component and molecular function. * GO with corrected *p*-value < 0.05 significantly enriched in DEGs.

The DEGs were characterized by using the Gene Ontology (GO) knowledgebase (http://geneontology.org/; accessed on 13 April 2020) to differentiate cellular, molecular, and biological functions between the two genotypes. GO enrichment bar charts show the top 30 enriched functions for up- and downregulated DEGs in the leaf (Figure 3) and root (Figure 4) tissues of PI 175311. The molecular functions (MF) included pectinesterase activity (GO:0030599), various enzymatic activities (GO:0003824, GO:0016788, GO:0016787, GO:0052689, and GO:0004553), and antiporter activity (GO:0015297); among the biological processes (BP) related to cell wall organization, structure, modification (GO:0042545, GO:0071555, GO:0045229, and GO:0071554), and membrane transport (GO:0055085, GO:0044765, and GO:1902578), along with cell wall and related external encapsulating structures (GO:0030312, GO:0005618, and GO:0005578) among cellular components (CC) were enriched in the upregulated unigenes in leaf tissues (GO with corrected *p*-value < 0.05 was significantly enriched in DEGs). On the other hand, only the biological processes related to fatty acid metabolism (GO:0006633, GO:0006631), monocarboxylic acid biosynthesis (GO:0072330), and protein phosphorylation (GO:0006468) were enriched among the downregulated DEGs in leaf tissues. Although none of the GO terms were enriched explicitly in the DEGs upregulated in root tissues, biological processes related to response to stress (GO:0006950), oxidative stress, and the oxidation–reduction process (GO:0006979, GO:0055114), as well as several molecular functions associated with oxidoreductase activities (GO:0016491, GO:0016684), peroxidase activity (GO:0004601), and heme- (GO:0020037) and tetrapyrrole (GO:0046906)-binding functions, were enriched in the downregulated DEGs in root tissues.

**Figure 5:**
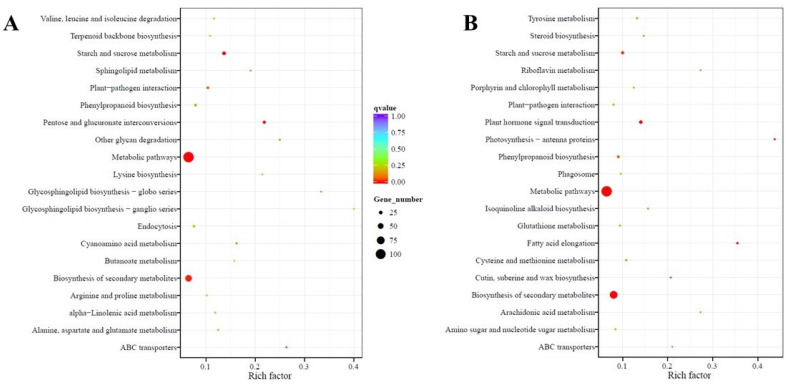
Statistics of pathway enrichment: The KEGG enrichment scatter plot analysis shows enriched pathways for (**A**) up and (**B**) downregulated differentially expressed genes (DEGs) in leaf tissue of PI 175311. The Rich factor is the ratio of DEGs in this pathway term to all gene numbers annotated in this pathway term. A q-value is the corrected *p*-value ranging from 0 to 1, and a lower value indicates greater pathway enrichment. The pathway names are shown on the *y*-axis, rich factor on the *x*-axis, size of the point represents the number of DEGs, and the color of the dot represents the q-value.

**Figure 6:**
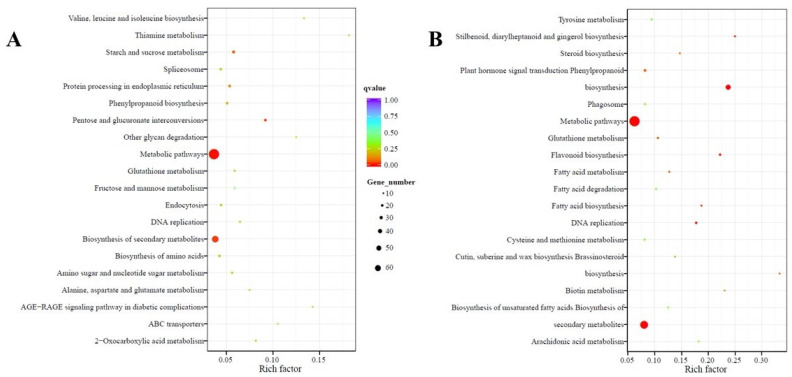
Statistics of pathway enrichment. The KEGG enrichment scatter plot analysis shows enriched pathways for (**A**) up and (**B**) downregulated differentially expressed genes (DEGs) in root tissue of PI 175311. The Rich factor is the ratio of DEGs in this pathway term to all gene numbers annotated in this pathway term. A q-value is the corrected *p*-value ranging from 0 to 1, and a lower value indicates greater pathway enrichment. The pathway names are shown on the *y*-axis, rich factor on the *x*-axis, size of the point represents the number of DEGs, and the color of the dot represents the q-value.

The Kyoto Encyclopedia of Genes and Genomes (KEGG) pathway database was used to determine the significantly enriched pathways for DEGs in this study. The KEGG enrichment analyses showing the top 20 enriched pathways in up- and downregulated DEGs in leaf and root tissues are shown in Figure 5 and Figure 6, respectively. The enriched pathways represented most plant metabolic pathways for compounds involved in biosynthesis, catabolism, utilization, assimilation, detoxification, and generation of precursor metabolites (Table S9), indicating their role in the regulation of oxalate metabolism in spinach. Pathways such as “metabolic pathways” and “biosynthesis of secondary metabolites” were enriched in both up- and downregulated DEGs in the leaf and root tissues of PI 175311, suggesting their involvement in regulating oxalate metabolism. The pathways related to “starch and sucrose metabolism” involved in the synthesis of D-glucose, the primary substrate for ascorbate synthesis [49], and “ABC transporters” were also enriched in upregulated DEGs in leaf tissues, while “pentose and glucuronate interconversions” involved in ascorbate synthesis were enriched in upregulated DEGs of both leaf and root tissues. The role of the galacturonate pathway in contributing to the ascorbate pool has been validated in plants [50,51,52].

### 2.5. Enrichment Analysis of Transcription Factors (TFs)

We performed TF enrichment analysis using PlantRegMap [48] in order to understand the possible regulatory mechanisms of oxalate metabolism in spinach. TFs possessing significantly overrepresented targets in the DEGs of both of the genotypes were identified. TF enrichment analysis identified 102 and 85 TFs possessing significantly overrepresented targets in the up- and downregulated DEGs, respectively, in the leaf tissues (Table S10; Supplementary Materials), and 47 and 72 TFs in the up- and downregulated DEGs, respectively, in the root tissues (Table S11; Supplementary Materials). The 625 upregulated and 958 downregulated putative target genes in leaf tissues had associated GO annotation, and were enriched for 70 and 71 GO terms, respectively, spread across several biological processes (Table S12; Supplementary Materials). Similarly, 320 upregulated and 627 downregulated putative target genes in root tissues had associated GO annotation, and were enriched for 71 and 147 GO terms, respectively, spread across several biological processes (Table S13; Supplementary Materials). Among the total TFome, the families of enriched TFs showing predicted interactions with the upregulated DEGs were overrepresented by the NAC and bZIP families, while for downregulated genes, MYB and TCP were prominent. For root tissues, TFs belonging to the Dof and WRKY families were overrepresented for up- and downregulated genes, respectively.

### 2.6. Quantitative Real-Time PCR Validation of DEGs from RNA-Seq

**Figure 7:**
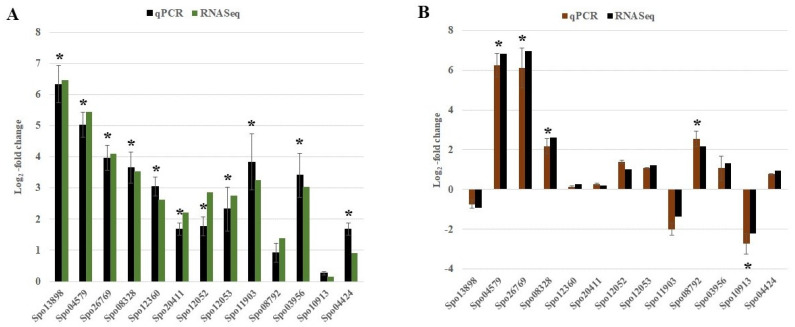
Fold changes (Log2) in the gene expression by qPCR and RNA-Seq analysis. Fold change (Log2) in the expression profiles of selected genes (including genes involved in the oxalate pathway) in (**A**) leaf and (**B**) root tissues of PI 175311. The error bars are the mean ± SE (*n* = 3); asterisks (*) represent significant differences between genotypes (*p* < 0.05) for qPCR analysis.

To validate the RNA-seq results, we selected 13 DEGs to confirm their expression patterns using quantitative real-time PCR. The fold changes of the selected genes using RT-qPCR were consistent (Figure 7) with the results obtained via RNA-Seq analysis (R^2^ = 0.90 for leaf and R^2^ = 0.97, for root), indicating the reproducibility and reliability of the RNA-seq data (Figure S5; Supplementary Materials).

## 3. Discussion

Oxalic acid, an ubiquitous dibasic acid in plants, is actively involved in several processes regulating abiotic metal stress, ion balance, and insect defense. Oxalic acid is a reducing agent, and its conjugate base chelates many divalent cations—such as Ca, Mg, zinc (Zn), manganese (Mn), and iron (Fe)—forming oxalate. Ca oxalate is the primary form found in spinach [32]. Among the micronutrients, there were significant increases in Ca, Mg, and K in high-oxalate PI 175311 (Table S1; Supplementary Materials), consistent with the significance of oxalate in calcium regulation and ion balance [38]. The role of oxalate in sequestering excess Ca^2+^ by storing large amounts in vacuoles in the form of Ca oxalate crystals—thereby prohibiting increases in Ca^2+^ levels in other compartments and maintaining the concentration gradient between the organelles—has been proposed [5,53]. The higher total nitrogen level in PI 175311 is consistent with a previous report [27] showing higher nitrate accumulation, along with high oxalate levels.

The RNA-Seq data analysis identified a set of up- and downregulated DEGs in both sets of tissues, which improved our understanding of the underlying molecular mechanisms associated with contrasting oxalate phenotypes. No significant changes in the photosynthetic performance in both genotypes were consistent, with the results showing no such changes in rice plants accumulating different oxalate levels [54]. Although no previous studies have identified coordinated transcriptional regulation of the genes associated with oxalate metabolism in plants, the subset of TFs identified in this study could help identify target genes for the manipulation of oxalate content in spinach or other plant species. Although analysis of the expression patterns of photorespiratory genes using a bioinformatic approach suggested strong co-expression, and conserved cis-elements in their 5′ upstream regions [55], this does not necessarily suggest co-regulation by common TFs. Several studies have identified transcription factors that conditionally regulate individual genes associated with intrinsic processes such as photorespiration, the glyoxylate cycle, or ascorbate metabolism. For example, the transcription of rice *ICL* is regulated by *WRKY71* during salt-stress-induced senescence or ABA treatment [56].

Several pathways for oxalate production through the cleavage of isocitrate, hydrolysis of oxaloacetate, oxidation of glycolate/glyoxylate, and cleavage of ascorbic acid have been proposed in plants [28]. However, a clear picture confirming the significance of these pathways in oxalate metabolism in spinach has not yet emerged. The validation of the contrasting oxalate phenotype established a framework for the understanding of the molecular regulation of oxalate metabolism under normal physiological conditions in spinach. Here, the relative contribution of genes associated with pathways involved in oxalate synthesis or catabolism is discussed based on the differential gene expression profiles of plants with contrasting oxalate phenotypes.

### 3.1. Oxalate Biosynthesis

#### 3.1.1. Cleavage of Isocitrate

In plants, glycolate and glyoxylate, which serve as precursors for oxalate synthesis, are produced through photorespiratory and photorespiration-independent metabolic reactions. An alternate route to photorespiration that compensates for glyoxylate formation via anaplerotic reactions has been validated in rice [39]. Glyoxylate is also produced by isocitrate via isocitrate lyase (ICL), a key enzyme involved in the glyoxylate cycle. The forms of the ICL enzyme in the germinating oil-storing seeds are different from those in green leaves, where it interconverts glyoxylate, succinate, and isocitrate [57]. The RNA-seq analysis revealed a six-fold (Log_2_) upregulation in the expression of *ICL* (Spo13898) in the high-oxalate-accumulating leaves of PI 175311 (Figure 7; Table S5). The role of *ICL* in oxalate synthesis has been demonstrated in tobacco [58] and *Rumex obtusifolius* [59]. Consistent with our results, overexpression of *ICL* in rice led to increased oxalate synthesis [39] and upregulation of *ICL* and malate synthase (*MLS*) in the absence of functional glycolate oxidation [60]. Furthermore, the concurrent upregulation of genes (Table S5) involved in fatty acid oxidation—such as fatty acid beta-oxidation multifunctional protein (MFP2; Spo21149); acyl-coenzyme A oxidase (Spo01759); and 3-hydroxy acyl-CoA dehydrogenase (Spo21149), required to generate acetyl-CoA for the glyoxylate cycle—are consistent with increased *ICL* expression in PI 175311. It has been demonstrated that oxalate accumulation positively correlates with increasing amounts of nitrogen, especially NO_3_, in spinach [20,21,22,24,25,26,27]. Previously, we analyzed transcriptomic responses in leaf and root tissues in response to nitrogen perturbation in spinach [61]. Consistent with the upregulation of *ICL* in PI 175311 in this study, high NO_3_ treatment also resulted in the upregulation of *ICL* in leaf (3.3-fold log_2_ scale) and root tissues (1.45 fold Log_2_ scale). Taken together, ICL seems to play a pivotal role in oxalate biosynthesis in spinach.

#### 3.1.2. Hydrolysis of Oxaloacetate

The ICL form present in green leaves interconverts glyoxylate, succinate, and isocitrate [53,57], as well as the formation of oxaloacetate, which could serve as a precursor for oxalate synthesis. The conversion of oxaloacetate to oxalate is catalyzed by oxaloacetate acetylhydrolase, or oxaloacetase (OAH/OXAC, EC 3.7.1.1), in *Ascomycota* fungi [62]. However, in the absence of detailed kinetic studies, the functional significance of *OXAC* genes to oxalate metabolism in plants remains to be confirmed. Nevertheless, such activity has been reported only once in the crude preparations of beetroot and spinach almost five decades ago [63]. In contrast with the previous report [31], our RNA-Seq data did not show induced expression of any of the three putative *OXAC* genes in high-oxalate PI 175311. However, upregulation of putative oxaloacetate acetylhydrolase (*OXAC2*, Spo21624) in PI 175311 roots (Table S7) can in part explain its tissue-specific activity and high oxalate accumulation. Given the increased accumulation of oxalate in response to NO_3_, our previous study showing upregulation of *OXAC2* in the spinach leaf tissue in response to high nitrogen level [61] suggests the possible role of OXAC-mediated oxalate biosynthesis via oxalacetate only under specific conditions. A concomitant increase in most organic acid intermediates (malate, citrate, 2-oxoglutarate, and succinate) along with oxalates in the TCA (tricarboxylic acid) cycle in response to high nitrates further substantiated the role of oxaloacetate in oxalate synthesis in spinach [64]. On the other hand, non-overlapping upregulation of different isoforms (*OXAC1* and *OXAC3*) in response to NO_3_ [31] may also indicate differential tissue specificities and genotypic differences.

#### 3.1.3. Oxidation of Glycolate/Glyoxylate

No significant differences in the photosynthetic rate or other gas analyzer measurements between high-oxalate PI 175311 and Bloomsdale were observed (Table S2), possibly implying the minimal contribution of photorespiratory intermediates to oxalate synthesis. Glycolate oxidase (GLO/GOX) catalyzes the conversion of photorespiratory glycolate to glyoxylate, and glyoxylate to oxalate, by utilizing its side reaction [53]. Unlike the previous report [31] that showed increased expression of putative *GLO* genes in the leaf tissues of high-oxalate lines, RNA-Seq analysis did not show differential expression of any of the five spinach *GLO* genes, suggesting the presence of alternative routes of glyoxylate synthesis. Conditions that altered photorespiration, such as high light intensity and high CO_2_ [39], or mutation in *GLO* [44], did not change oxalate levels, further confirming the occurrence of photorespiration-independent oxalate synthesis. Increased expression of *ICL* in PI 175311 in this study suggests that in the absence of photorespiration, the glyoxylate cycle provides anaplerotic reactions to compensate for glyoxylate formation. Nevertheless, GLO side reactions can still participate in oxalate synthesis by catalyzing excess glyoxylate. The expression of another photorespiratory-cycle-related putative mitochondrial alanine–glyoxylate aminotransferase (Spo12360) involved in the detoxification of glyoxylate was upregulated in PI 175311 leaves (Table S5). Toxic effects of excess glyoxylate on inhibiting RuBP regeneration have been demonstrated in spinach chloroplasts [65]. Although the glyoxylate and glycolate shuttling across the organs is tightly regulated during photorespiration in plants, it has been suggested that the excess glyoxylate leaking out from peroxisomes can be rescued by glyoxylate reductase (*GLY/GR*) by reducing it to glycolate [66,67]. In PI 175311, strong upregulation of three glyoxylate/succinic semialdehyde reductase-like genes (Spo04579, Spo00830, and Spo26769) was observed (Table S5), of which the former two are truncated proteins, while the latter shared over 64% homology with Arabidopsis GLY1. The presence of specific activities of NADPH-dependent GLY isoforms in the cytosol and chloroplasts have been characterized in spinach [68] and Arabidopsis [69,70]. As GLYRs are expected to play a role in the detoxification of glyoxylate pools, it is not well understood how their specificities and localization could impact oxalate synthesis in plants. Although there is not enough evidence to support the production of glycolate or glyoxylate via Rubisco or the glyoxylate cycle in spinach roots, the expression of *GLO* genes in spinach roots, as in Arabidopsis [71], suggests the possibility of localized synthesis using transported substrates. Long-distance translocation of oxalate from source to sink has been suggested in rice [72].

#### 3.1.4. Oxidative Cleavage of Ascorbic Acid

The oxidative degradation of ascorbate involves cleavage of the C_2_–C_3_ bond of L-ascorbic acid to L-threonate, which is converted to oxalate. Several studies have demonstrated oxalate synthesis via ascorbate degradation [5,73,74,75], although the enzymes responsible for this pathway have not been functionally characterized in plants. RNA-seq analysis identified strong upregulation (3.5-fold log_2_) of the most abundant (based on FRKM) ascorbate peroxidase (*APX*; Spo08328), as well as two highly expressed ascorbate oxidases (*AO*; Spo12052 and Spo12053), suggesting increased degradation of ascorbates in PI 175311 (Table S5). In plants, both APX and AO enzymes are involved in oxidizing ascorbates to produce dehydroascorbic acid (DHA), converted to oxalate via a series of enzymatic and non-enzymatic reactions [76]. Production of oxalate, threonic acid, and oxalyl-L-threonates as end products of ascorbate degradation has been demonstrated in tomatoes [75]. The synthesis of oxalate from ascorbate was validated long ago in spinach seedlings using radiolabeled precursors [77]. A recent study that monitored the fate of ascorbate degradation during the postharvest handling of spinach leaves using radiolabeled [^14^C] ascorbate provides direct evidence of oxalate synthesis through ascorbate [78].

### 3.2. Oxalate Degradation

Oxalate catabolism in plants proceeds via three routes: CoA-dependent decarboxylation, oxalate-oxidase-mediated oxidation, and oxalate acetylation. [79,80]. Oxalate decarboxylase (OXDC) activity catabolizing oxalate to formate and CO_2_ has not been demonstrated in plants, although heterologous overexpression of fungal *OXDC* reduced the accumulation of oxalate in tomato fruits [81]. The previously reported [31] putative *OXDC* (*Spo06441, Spo19759,* and *Spo25084*)-like genes in spinach share poor homology with known fungal *OXDC* genes, suggesting the need for the functional validation of spinach enzymes in order to ascertain the significance of OXDC in oxalate degradation. The absence of any significant changes in the expression of these genes in the current study suggests a subordinate role of decarboxylation in oxalate catabolism. Manipulation of plant oxalate content through the activation of oxalate catabolic genes has been suggested [79,82]. Germin-like oxalate oxidase (GXO) activity that converts oxalate to CO_2_ and H_2_O_2_ has been detected in monocots [83,84], but not in dicots [85,86]. Despite the relatively high expression of putative GXO/OXO-like genes reported in spinach leaf tissues [31], the corresponding enzyme activities have not been detected in spinach [87]. The RNA-Seq analysis did not show any change in the expression of the *OXO* genes in the leaf. The expression of putative *OXO2* in PI 175311 root tissues showed a trend for downregulation, although this was not statistically significant (q-value = 0.10), suggesting the role of *OXO* in regulating oxalate accumulation in roots by suppressing its catabolism. Previously, two oxalate-oxidase-like germins (165 and 172) with possible roles in nitrate stress have been reported in spinach roots [88]. The acetylation pathway is mediated by four enzymes: oxalyl-CoA synthase, oxalyl-CoA decarboxylase, formyl-CoA hydrolase, and formate dehydrogenase. The first enzyme in this pathway—acyl-activating enzyme 3 (*AAE3*)—involved in oxalate acetylation, has been characterized in Arabidopsis [79] and other plants [82,89]. Consistent with previous studies [31], our qPCR analysis also showed a significant upregulation (*p*-value < 0.05) of spinach *AAE3* (Spo04424) in PI 175311 leaf and root tissues (Figure 7), and a trend for upregulation in the RNA-Seq data (q-value = 0.07) (Table S2). Recent studies characterizing downstream enzymes involved in the degradation of oxalyl-CoA support the importance of the acetylation pathway in oxalate regulation in plants [90,91]. The induction of *AAE3* in roots to avoid the harmful effects of oxalate in response to aluminum stress [92] is consistent with its higher expression in PI 175311, suggesting its role in oxalate homeostasis.

## 4. Conclusions

The current study demonstrated that the transcriptomic analysis of spinach tissues with contrasting oxalate phenotypes, under normal physiological conditions, indicates a complex network of genes associated with intrinsic metabolic and physiological processes that regulate oxalate homeostasis in spinach. The RNA-Seq analysis identified a total of 2308 leaf-specific and 1686 root-specific DEGs, suggesting several molecular mechanisms that define the high-oxalate genotype. GO analysis of DEGs identified molecular functions associated with various enzymatic activities and biological processes associated with transport across membranes in the upregulated genes, along with fatty-acid-metabolism-related processes in the downregulated DEGs in leaf tissues. KEGG pathway analysis revealed enrichment of the metabolic and secondary metabolite pathways. The expression profiles of genes associated with distinct physiological processes suggest that the glyoxylate cycle, ascorbate catabolism, and photorespiratory pathway may collectively regulate oxalate in spinach. The data indicate a pivotal role of isocitrate lyase in oxalate synthesis in spinach. Alternative pathways through ascorbate or oxaloacetate degradation, utilizing photorespiratory intermediates, may also contribute to maintaining steady levels of oxalate under certain conditions. The findings from this study provide the foundation for novel insights into the broad-spectrum oxalate metabolism in plants.

## 5. Materials and Methods

### 5.1. Plant Growth and Estimation of Oxalates in Spinach

The spinach plants were grown in an environmentally controlled growth chamber at the Texas A&M AgriLife Research and Extension Center, Uvalde, TX, USA. The seeds of spinach accession PI 175311 (the North Central Regional Plant Introduction Station, USDA-ARS, Iowa State University, Ames, IA, USA) and the Bloomsdale variety were grown inside a controlled growth chamber in plastic containers containing Turface^®^-based growing medium under 200 μmol·m^−2^·s^−1^ light intensity (12 h each light and dark periods) at 23 °C and 60–70% relative humidity. The plants were fertilized using N-free Hoagland’s nutrient solution (No. 2 Basal Salt Mixture HOP03-1LT, Caisson Labs, Smithfield, UT, USA). The leaf and root samples collected from 6-week-old plants were frozen in liquid nitrogen and stored at −80 °C until subsequent analyses. Three independent plants were used for metabolich analysis, total RNA extractions, and physiological and biochemical analysis.

### 5.2. Estimation of Photosynthesis, Minerals, and Quantification of Free Amino Acids

The leaf photosynthetic rate (Pn), transpiration rate (Tr), stomatal conductance (Cs), and intercellular CO_2_ concentration were measured using a LICOR-6400XT Portable Photosynthesis System (Lincoln, NE, USA), from the fully expanded leaves of 6-week-old plants. The chlorophyll content was measured using a portable chlorophyll meter (SPAD-502, Konica Minolta, Tokyo, Japan). The plant samples were analyzed for total N (TKN), NO_3_^−^, and NH_4_^+^ using a Rusing EasyChem Plus analyzer (Chinchilla Scientific, Oak Brook, IL, USA). The amino acid analysis was performed using a Waters^TM^ Acquity H-class UPLC system coupled with a Waters^TM^ Xevo TQs MS–MS mass spectrometer with an electrospray ionization (ESI) probe following a pre-established protocol [93]. Data integration and quantitation were carried out using Waters’ TargetLynx ™ software. The statistical program JMP Pro 14 (SAS Institute, Cary, NC, USA) was used for statistical analysis.

### 5.3. Oxalic Acid Estimation

Oxalate content was then determined using an oxalate kit (KA4532; Abnova, Taipei, Taiwan). Approximately 15 mg of leaf tissue was homogenized in liquid nitrogen using a paint shaker and then extracted using ice-cold assay buffer, as per the manufacturer’s protocol. Using this method, in the presence of the “oxalate convert” and “oxalate enzyme mix”, oxalate forms an intermediate that reacts with a particular probe to generate color at 450 nm. Oxalate standards were prepared in a 96-well flat-bottom microplate in order to establish a standard curve (0–10 nmol). The amount of oxalate in the sample wells was determined using a multi-well spectrophotometer (Multiskan GO microplate spectrophotometer, Thermo Scientific, Waltham, MA, USA) and comparing absorbance to the standard oxalate curve. The oxalate concentration in spinach leaves was calculated as mg/100 g of fresh weight for each sample.

### 5.4. RNA-Seq Analysis of Spinach Leaves and Roots

Twelve independent libraries were created using total RNA samples from three replicates of leaf and root tissues from PI 175311 and Bloomsdale. The samples were flash-frozen in liquid nitrogen and ground to a fine powder using 3-mm-diameter steel balls (Abbott Ball, West Hartford, CT, USA) in a paint shaker (Harbil, Wheeling, IL, USA). Total RNA was extracted using an RNeasy^®^ Plant Mini Kit (QIAGEN Sciences, Germantown, MD, USA), as per the manufacturer’s protocol. The purity of the RNA was confirmed using the NanoPhotometer^®^ spectrophotometer (IMPLEN, CA, USA). The RNA Nano 6000 Assay Kit of the Bioanalyzer 2100 system (Agilent Technologies, Santa Clara, CA, USA) was used to assess RNA integrity and quantitation. Sequencing libraries were generated using the NEBNext^®^ Ultra™ RNA Library Prep Kit for Illumina^®^ (NEB, Ipswich, MA, USA), following the manufacturer’s protocol. The clustering of the index-coded samples was performed on a cBot Cluster Generation System using a PE Cluster Kit cBot-HS (Illumina), according to the manufacturer’s instructions. After cluster generation, the libraries were sequenced on an Illumina Hiseq platform, and 150 bp paired-end reads were generated. Raw reads of fastq format were processed in order to obtain clean reads by removing the adapter, reads containing poly N (reads when uncertain nucleotides constitute more than 10 percent of either read; N > 10%), and low-quality reads (reads when low-quality nucleotides (base quality less than 20) constitute more than 50% of the read. The Q score (Quality value) of over 50% bases of these read is ≤5) from the raw data. At the same time, the Q20, Q30, and GC content of the clean data were calculated. Spinach reference genome and gene model annotation files were downloaded from SpinachBase (http://spinachbase.org/, accessed on 13 April 2019). The index of the reference genome was built using Bowtie v2.2.3, and paired-end clean reads were aligned to the reference genome using TopHat v2.0.12. HTSeq v0.6.1 was used to count the reads mapped to each gene. The FPKM [94] of each gene was calculated based on the length of the gene and the number of reads mapped to the gene. Differential expression analysis of the genes was performed using the DESeq R package (1.18.0) [95]. Genes with a q-value (padj) (*p*-value after normalization) < 0.05 found by DESeq were considered to be differentially expressed. Gene Ontology (GO) [96] enrichment analysis of differentially expressed genes was implemented using the GOseq R package, with which gene length bias was corrected. GO terms with *p*-values of less than 0.05 were considered to be significantly enriched by DEGs. KOBAS software in the Kyoto Encyclopedia of Genes and Genomes (KEGG) pathway database [97] was used to test the statistical enrichment of differential expression genes. The online tool Venny v.2.0.2 [98] was used to show the distribution of differentially expressed genes using Venn diagrams. The RNA-Seq dataset is accessible through the GEO Omnibus (https://www.ncbi.nlm.nih.gov/geo, accessed on 10 May 2021) database (Series GSE146711). The transcription factor (TF) enrichment analysis and the interaction between TFs and their target genes was evaluated using PlantRegMap [48], and the number of TFs with significantly overrepresented target number genes was retrieved (*p*-value < 0.01; Fisher’s exact test).

### 5.5. Validation by Quantitative Real-Time PCR

The expression patterns of 13 selected DEGs, based on their significance in RNA-Seq analysis, were examined using quantitative real-time PCR (RT-qPCR) in both tissue types. The gene-specific primers based on the selected unigene sequences (Table S14; Supplemental Materials) were designed using Primer Premier 3.0 software. Total RNA was extracted using an RNeasy^®^ Plant Mini Kit (QIAGEN Sciences, Germantown, MD, USA), as per the manufacturer’s protocol, followed by treatment with DNase1 (QIAGEN Sciences, Germantown, MD, USA), and was then subjected to reverse transcription using iScript RT Supermix (Bio-Rad Laboratories, Inc, Hercules, CA, USA). The quality and quantity of the RNA were examined using a Denovix DS-11+ spectrophotometer (Wilmington, DE, USA). Gene expression analysis via reverse transcription-qPCR was performed using a BioRad CFX96 qPCR instrument and an SsoAdv Univer SYBR GRN Master Kit (Bio-Rad Laboratories, Inc, Hercules, CA, USA). The spinach 18S rRNA was used as internal control, and the relative expression levels (Cq values) of each gene were normalized by taking an average of three biological replicates. The relative expression levels of each gene were calculated using the 2−ΔΔCt method [99].

## Figures and Tables

**Table 1:** Oxalate content (mg/100 g) in leaves and roots of PI 175311 and Bloomsdale (*n* = 5; Mean ± Std Error).

	Fresh wt			Dry Wt.		
Tissue	PI 175311	Bloomsdale	*p*-Value	PI 175311	Bloomsdale	*p*-Value
Leaves	1312.9 ± 85.0	785.5 ± 89.2	0.0037	104.4 ± 5.7	54.4 ± 3.1	0.0287
Roots	450.0 ± 11.5	287.1 ± 33.3	0.0347	40.2 ± 3.0	20.8 ± 2.6	0.0347

## Data Availability

The RNA-sequencing data are available from the GEO (https://www.ncbi.nlm.nih.gov/geo, accessed on 10 May 2021) database (Series GSE146711).

## Supplementary Materials

The following are available online at https://www.mdpi.com/article/10.3390/ijms22105294/s1.
